# ID 351 - Produtos de ATS: desafios e possibilidades em um hospital universitário no sertão de Pernambuco

**DOI:** 10.5327/2237-9622.2025.v34s1.351

**Published:** 2025-11-25

**Authors:** Emanuela Oliveira Spinola, Thais Ferreira Lopes Diniz Maia, Paula Andreatta Maduro, Izabelle Silva de Araújo, Carine Rosa Naue, Orlando Vieira Gomes, Helder Nunes Lopes, Fabio Oliveira Lima, Osana dos Santos Cardoso, Raimundo Campos Palheta, Ricardo Santana de Lima

**Keywords:** Avaliação de Tecnologias em Saúde, Sistema Único de Saúde, hospital universitário, prática em saúde baseada em evidências

## Abstract

**Introdução:**

No Brasil, o Ministério da Saúde (MS) considera tecnologias em saúde: "medicamentos, equipamentos e procedimentos técnicos, os sistemas organizacionais, informacionais, educacionais e de suporte e os programas e protocolos assistenciais por meio dos quais a atenção e os cuidados com a saúde são prestados à população" (Ministério da Saúde, 2005. p. 1). As inovações tecnológicas são componentes essenciais dos sistemas de saúde que devem garantir o acesso equitativo às tecnologias com qualidade, segurança, eficácia e custo-efetividade (WHO, 2007). O constante avanço científico-tecnológico tem propiciado o desenvolvimento de inovações tecnológicas em saúde, cuja Avaliação de Tecnologias em Saúde (ATS) assume um lugar de destaque, tendo em vista o relevante papel de apoiar o processo de tomada de decisão dos gestores na incorporação ou desincorporação de tecnologias nos serviços de saúde, sobretudo no cenário de escassez de recursos com volume cada vez maior de demandas para os serviços do Sistema Único de Saúde (SUS). Para tanto, o Núcleo de Avaliação de Tecnologias em Saúde (Nats) constitui-se enquanto protagonista desse processo, cuja atuação consiste na sistematização de evidências científicas rigorosas e auditáveis que respaldem a decisão contribuindo para o acesso às tecnologias. O Nats em questão tem dois anos de estruturação, pertencente a um hospital universitário (HUF) da Empresa Brasileira de Serviços Hospitalares (Ebserh), localizado no sertão de Pernambuco, cuja experiência suscitou a questão "Quais os desafios e as possibilidades na sistematização dos produtos das ATS?". O objetivo deste resumo é refletir sobre desafios e possibilidades na sistematização dos estudos de ATS e na elaboração dos documentos ou produtos de um Nats no sertão de Pernambuco.

**Método:**

Trata-se de estudo de natureza qualitativa, pesquisa tipo exploratória e interdisciplinar com enfoque na revisão bibliográfica e documental (produtos das sínteses de evidências científicas de domínio público) realizada durante dois anos de atuação de um Nats no sertão de Pernambuco.

**Resultados:**

Foram vivenciados três processos de ATS, envolvendo tecnologias diferenciadas (medicamento e equipamentos), todos avaliados em duplas, documentos produzidos avaliados e deliberados de forma colegiada, os quais fundamentaram a tomada de decisão da gestão e a posterior publicação em repositório website do Nats.

**Conclusão:**

Os dados apontam para uma trajetória de desafios relacionados ao desconhecimento e à inexperiência em ATS, bem como no uso de suas ferramentas/instrumentos, e à inexistência de carga horária protegida para os membros. Entretanto, a particularidade de o Nats estar vinculado a um HUF também favoreceu oportunidades: qualificação, identificação, aproximação e estabelecimento de parcerias estratégicas com a universidade, desenvolvimento de habilidades e competências intrínsecas ao processo de ATS, troca com projetos de iniciação científica e tecnológica e grupo de pesquisas em práticas baseadas em evidências. O diferencial nesse processo é fazer parte de duas redes – Nats Ebserh e Rede Brasileira de Avaliação em Tecnologias em Saúde (Rebrats) –, pois isso tem possibilitado a qualificação de forma mais estruturante e o fortalecimento dos Nats para que de fato possam contribuir para o acesso da população, de forma equitativa e segura, às tecnologias em saúde, materializando assim sua função social.

